# Hypothalamic-Pituitary Axis Dysfunction, Central Diabetes Insipidus, and Syndrome of Inappropriate Antidiuretic Hormone Secretion as the First Clinical Presentation of Neurosarcoidosis: Why Early Diagnosis and Treatment is Important?

**DOI:** 10.7759/cureus.11481

**Published:** 2020-11-14

**Authors:** Tatjana Blazin, Dhruvil Prajapati, Linha (Lina) M Mohammed, Meera Dhavale, Mohamed K. Abdelaal, A B M Nasibul Alam, Natalia P Ballestas, Jihan A Mostafa

**Affiliations:** 1 Research, California Institute of Behavioral Neurosciences & Psychology, Fairfield, USA; 2 Psychiatry, California Institute of Behavioral Neurosciences & Psychology, Fairfield, USA

**Keywords:** neurosarcoidosis, central diabetes insipidus, syndrome of inappropriate antidiuretic hormone secretion, hypothalamo-pituitary sarcoidosis, antidiuretic hormone, sarcoidosis, antidiuretic hormone and neurosarcoidosis

## Abstract

Sarcoidosis is defined by granuloma formation in a multitude of organs. Despite its rare involvement in the nervous system, there are a number of cases that identify neurological symptoms to be the initial clinical manifestation of sarcoidosis. The involvement of the hypothalamic-pituitary (HP) axis presented most frequently with hormone deficiencies. Studies have reported that damage to the pituitary gland may be irreversible, and hormone abnormalities were generally permanent. Neurosarcoidosis has been described as the underlying cause of central diabetes insipidus (DI) and syndrome of inappropriate antidiuretic hormone (SIADH) secretion. The pathological mechanism that can lead both to deficiency and excess of antidiuretic hormone (ADH) secretion is still not fully understood. It has been shown that diagnosis of neurosarcoidosis remains challenging, as symptoms can be inconclusive and diagnostic tools are not sufficiently sensitive and specific. Early treatment may potentially reverse pituitary deficiencies, although studies to confirm this hypothesis are minimal. This review article aims to increase knowledge about central DI and SIADH caused by neurosarcoidosis, identify possible difficulties in diagnosis, and discuss the importance of early management. Clinical trials investigating the long-term therapeutic response in patients with HP sarcoidosis are essential, as there are currently no established guidelines for the treatment of neurosarcoidosis.

## Introduction and background

The symptoms of sarcoidosis are often dismissed by doctors as not important, and they (the patients) can be interpreted by family members as somebody who is just complaining. This can impact the quality of life.

Debbie Durrer, executive director of the Foundation for Sarcoidosis Research

Sarcoidosis is a systemic disease characterized by the formation of non-caseating granulomas in various organs. The estimated worldwide prevalence of the disease is 60 per 100,000 people, with the highest rates observed in African Americans at 35-80 per 100,000, whereas European Americans' incidence is approximately 3-10 cases per 100,000 [1]. Given that sarcoidosis mimics many other diseases, the number of people affected is much higher than reported [2]. Sarcoidosis imposes a considerable economic burden, with direct medical costs estimated at $1.3-$8.7 billion to commercial insurance companies, in addition to an indirect cost of $0.2-$1.5 billion in work loss due to sarcoidosis [3]. The most frequent age of onset of sarcoidosis is between 20 and 40 years of age. Although the etiology remains unclear, it is hypothesized that sarcoidosis could be immune-mediated, environmental, genetic, or ethnic related, all of which could have significant contributing factors in the disease's pathogenesis and prognosis [2].

**Figure 1:**
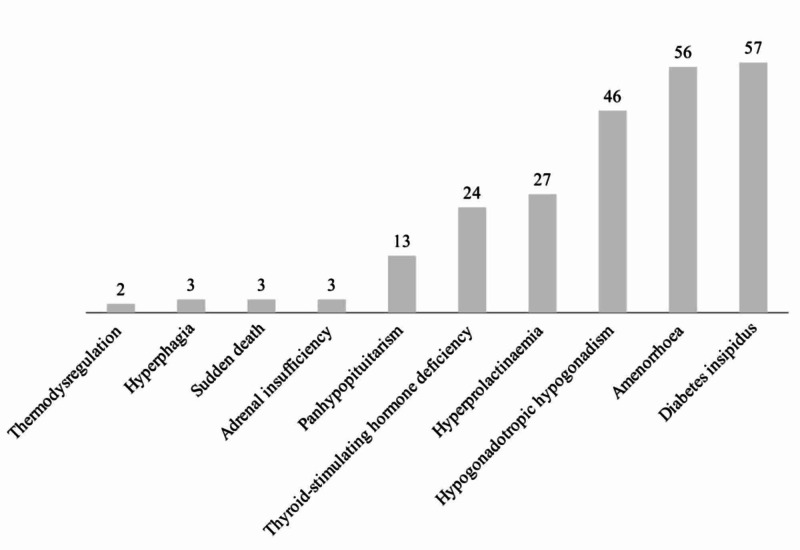
Incidence of neurosarcoidosis with endocrine dysfunction from 2002 to 2014

Neurosarcoidosis is a rare condition, occurring in approximately 5-15% of patients diagnosed with systemic sarcoidosis. Of those cases, 52% initially present with neurological symptoms [4,5]. Hypothalamic-pituitary (HP) involvement in neurosarcoidosis is often asymptomatic; however, it can present clinically in the form of endocrine dysfunction (Figure 1). The granuloma formation affecting HP may also lead to irreversible damage and hormone deficiencies [4-6]. However, this condition is rarely observed and has a prevalence of only 2.5% [7].

Brain magnetic resonance imaging (MRI) is the diagnostic study of choice due to its relatively high sensitivity at approximately 82-97% but lacks the specificity for diagnosing neurosarcoidosis [4,8]. Tissue biopsy is essential for definitive diagnosis, but it is not always the safest option; instead, a classification system which includes 'definitive,' 'probable' and 'possible' sarcoidosis is accepted. Therefore, the diagnosis of neurosarcoidosis remains challenging since it is typically based on supporting facts instead of pathohistological confirmation [6,8]. Moreover, endocrine changes primarily occur in previously undiagnosed cases of neurosarcoidosis, further reinforcing the importance of considering this condition as a differential diagnosis when evaluating the underlying reason for HP dysfunction. Correct and prompt diagnosis and treatment should be ensured since early treatment may salvage pituitary function [7-10].

An earlier study indicated that pituitary hormone abnormalities (PHA) were the first sign of systemic sarcoidosis in 54.3% of patients. Furthermore, PHA improvement despite treatment was reported in only 13% of patients with all-cause mortality of 8.7% [11]. The significant limitation of this study is that it included only four patients. Published cases over the last several years show that the initial manifestations of sarcoidosis may be panhypopituitarism, hyperprolactinemia, hypogonadotropic hypogonadism, thyrotropin dysfunction and amenorrhoea, hypothermia, syndrome of inappropriate antidiuretic hormone secretion (SIADH), and central diabetes insipidus (DI) [9,10,12-16]. The diagnosis of neurosarcoidosis was delayed in a substantial number of cases. Nevertheless, the diagnosis of neurosarcoidosis-induced DI may present an additional challenge as it can be masked by hypovolemia, hypothyroidism, and adrenal insufficiency [17-19].

This review article aims to highlight the most frequent neuroendocrine manifestations of sarcoidosis, particularly as an initial presentation of the disease with a focus on antidiuretic hormone (ADH) abnormalities. We also wanted to analyze the significance of sarcoidosis as a differential diagnosis when investigating hormonal imbalances. Furthermore, the necessity for more research regarding the diagnosis and treatment of neurosarcoidosis has been emphasized.

## Review

Neurosarcoidosis

Pathophysiology

Sarcoidosis is an inflammatory disease that presents with granuloma formation in multiple organs. Epitheloid granuloma formation begins with the interaction between dendritic cells, macrophages, T helper cells and occasional cytotoxic T lymphocytes, and B cells triggered by unclear immunologic mechanisms [2,20]. The clinical course and manifestations of sarcoidosis can be highly unpredictable. This condition mainly affects the lungs and lymph nodes of the thorax and the neck; however, skin, eyes, and the liver are commonly involved as well [2,5]. According to Caruana et al., sarcoidosis can be divided into acute sarcoidosis, chronic sarcoidosis with limited dissemination, chronic sarcoidosis with full dissemination, including cutaneous involvement, and chronic sarcoidosis with nervous system involvement [2].

Clinical Manifestations and Diagnosis

Neurosarcoidosis is a rare condition with various presentations depending on the part of the nervous system involved. The most frequent presentations of neurosarcoidosis are headache, cranial nerve dysfunction, ataxia, meningitis, sensory, or motor abnormalities (Table 1) [5,14]. An earlier study that included 82 patients described that neurosarcoidosis was the initial presentation in previously undiagnosed cases of sarcoidosis in 74% of patients, whereas the systemic review which included 622 patients described neurological symptoms as initial presentation in 52% of cases [1,5]. The American Thoracic Society, the European Respiratory Society, and the World Association of Sarcoidosis and Other Granulomatous Disorders (WASOG) indicated that while assessing the diagnosis for neurosarcoidosis three criteria must be fulfilled: typical clinical and radiological findings, noncaseating granulomas, and lack of evidence for the alternative disease [21]. However, diagnosis may be difficult because of nonspecific symptoms, the resemblance with other diseases, insufficient sensitivity and specificity of diagnostic tests, and variable diagnostic methods [22].

**Table 1:** Neurosarcoidosis manifestations CI: confidence interval.

Clinical manifestation	% of patients	95 CI
Cranial neuropathy	55%	52-58%
Facial nerve optic nerve	24%, 21%	21-27%, 18-24%
Headache	32%	28-35%
Sensory abnormalities	29%	24-33%
Motor symptoms	19%	15-22%
Hemiparesis paraparesis	9%, 11%	6-12%, 7-14%
Meningitis	16%	13-19%
Spinal cord abnormalities	18%	15-21%
Peripheral nervous system involvement	17%	14-21%
Myopathy	15%	9-11%

**Figure 2:**
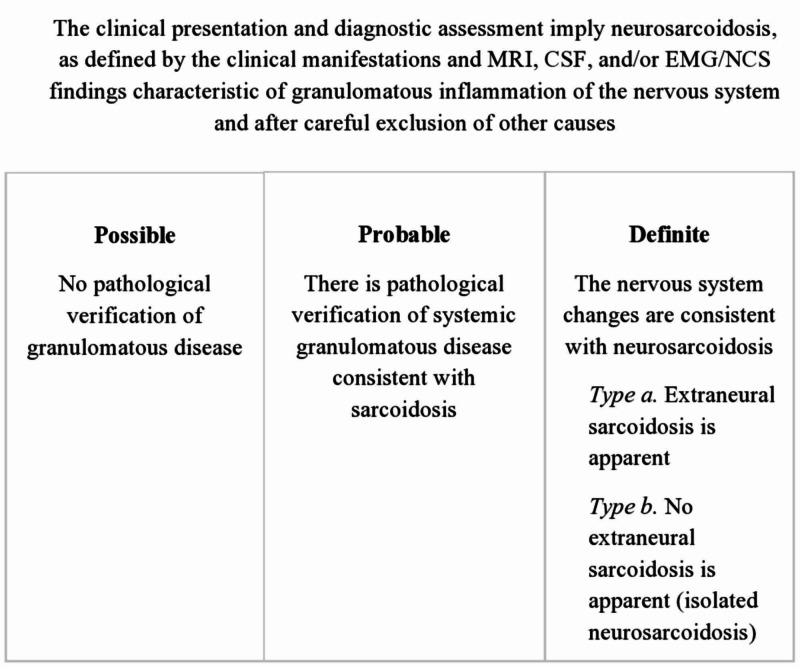
Criteria for the diagnosis of neurosarcoidosis Adapted from Stern et al. [8] MRI: magnetic resonance imaging, CSF: cerebrospinal fluid, EMG: electromyography, NCS: nerve conduction study.

Therefore, the Neurosarcoidosis Consortium Consensus Group developed an approach for diagnosing suspected neurosarcoidosis. According to these criteria, the diagnosis of ‘possible’, ‘probable’, and ‘definite’ neurosarcoidosis can be made (Figure 2) [8]. We can conclude that diagnosis of neurosarcoidosis remains complex knowing that the majority of patients lack a histological confirmation and diagnosis is made mostly according to the criteria aforementioned with most of the patients classified as ‘possible’ sarcoidosis [5].

Treatment

**Figure 3:**
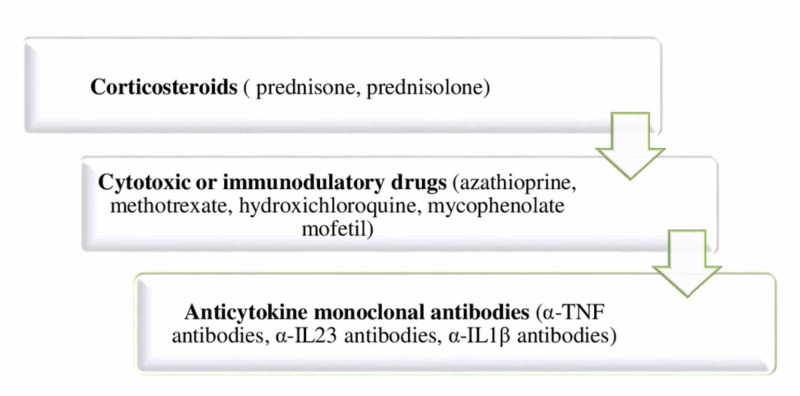
Treatment of neurosarcoidosis

In the case of neurosarcoidosis, treatment is always required and the treatment regimen depends on expert opinion, as well as on clinical manifestations. Glucocorticoids are generally the initial therapy, whereas the more severe cases are candidates for the second and third-line therapy which includes cytotoxic and immunomodulatory drugs, in addition to monoclonal antibodies (Figure 3) [22,23]. The prognosis of neurosarcoidosis varies in every case depending on the clinical course and severity of the disease [14]. As reported by the previous meta-analysis, complete remission in neurosarcoidosis is observed in 27% of patients, incomplete remission in 32%, stable disease in 24%, while deterioration and death occurred in 6% and 5%, respectively [5]. Therefore, despite proper therapy, there are still cases that do not improve or deteriorate with a considerable mortality rate. The clear guidelines for the treatment of neurosarcoidosis are essential, since adverse sequels may potentially be prevented.

Hypothalamic-pituitary axis dysfunction as a presentation of neurosarcoidosis

The HP axis involvement is an uncommon presentation of neurosarcoidosis. This condition can be asymptomatic and, in some cases, can be accidentally discovered while assessing hormonal levels [6]. However, granuloma affecting the HP axis can lead to various hormonal abnormalities. As reported in a previous multicenter study of 24 patients, 54% of patients had presented symptoms of pituitary dysfunction in previously undiagnosed cases of neurosarcoidosis. The same study showed that DI, hypogonadotropic hypogonadism, hyperprolactinemia, and thyroid-stimulating hormone deficiency can all be the first manifestations of HP sarcoidosis [24]. This is in line with the results of the study which demonstrated that endocrine dysfunction is most commonly the first symptom leading to the diagnosis of neurosarcoidosis [6]. Both studies agreed that central DI is present in a significant number of cases [6,24].

HP sarcoidosis can be defined based on clinical symptoms, hormonal deficiencies, MRI findings, or histopathology. This is the reason behind the complexity of estimating the real incidence of HP involvement in sarcoidosis [24]. A previous study determined that up to 2015, 155 cases of neurosarcoidosis with HP involvement have been described in the literature [6]. Furthermore, hypothalamic and/or pituitary lesions were found in 8.5% of patients diagnosed with neurosarcoidosis in an earlier cohort study from 2019 including 82 patients [1]. MRI findings in HP sarcoidosis most frequently include infiltrative lesions of the hypothalamus or pituitary gland and pituitary stalk thickening; however, normal MRI findings are reported in some cases which further can delay the diagnosis [1,4,24]. Despite the improvement in MRI findings after treatment, most hormonal deficiencies are permanent [24]. This reinforces the question of what can be done to prevent possible irreversible changes present in HP sarcoidosis.

Central diabetes insipidus in neurosarcoidosis

Pathophysiology

Central DI is caused by impairment of ADH secretion from the posterior pituitary gland. ADH, also called arginine vasopressin, is synthesized in the supraoptic and paraventricular nuclei of the hypothalamus. The projection of neurons from these nuclei to the posterior pituitary leads to the secretion of ADH in the systemic circulation. Following the ADH interaction with receptors in kidneys, ADH plays a crucial role in water homeostasis. Central DI is caused by neurohypophysial destruction and it is suggested that more than 90% of vasopressinergic neurons have to be damaged to cause symptoms of ADH deficiency [25,26]. Central DI due to neurosarcoidosis is described in a limited number of studies. It is important to highlight that symptoms of central DI were the first manifestation of sarcoidosis in a substantial number of reported cases. Out of 13 case reports included in this study, DI was the first clinical presentation of sarcoidosis in 10 cases. Two studies described the cases of DI in patients with previously diagnosed neurosarcoidosis, while in one case, the patient diagnosed with sarcoidosis presented with central DI as the first manifestation of neurosarcoidosis [16-19,27-37]. This observation falls within the range of a previously reported review article from 2000 that described five patients who all presented with symptoms of DI and subsequently were diagnosed with neurosarcoidosis [29]. Interestingly, two cases of 10- and 15-year-old girls presenting with symptoms of central DI as the first manifestation of sarcoidosis were also reported [35,36]. Therefore, central DI as the first presentation of neurosarcoidosis is recognized unexpectedly in the pediatric population as well.

Clinical Manifestations

The classic presentation of the disease includes symptoms of polyuria and polydipsia [16,27,28,31-34]. Moreover, hypothalamic lesions can lead to the loss of thirst sensation, therefore, cases of adipsic central DI have also been described [17,38]. In some cases, neuropsychiatric symptoms along with altered mental status were the first clinical manifestations of this condition [17,18]. Central DI due to neurosarcoidosis can be an isolated finding or can be concomitant with symptoms of panhypopituitarism, hypogonadotropic hypogonadism, secondary/tertiary thyroid insufficiency, secondary adrenal insufficiency, and hyperprolactinemia [27-29,31,33,34,37]. Laboratory investigations revealed hypernatremia and low urine osmolality in the majority of patients. It is estimated that approximately 2% of patients with neurosarcoidosis will have symptoms of central DI [38].

Diagnosis and Treatment

**Figure 4:**
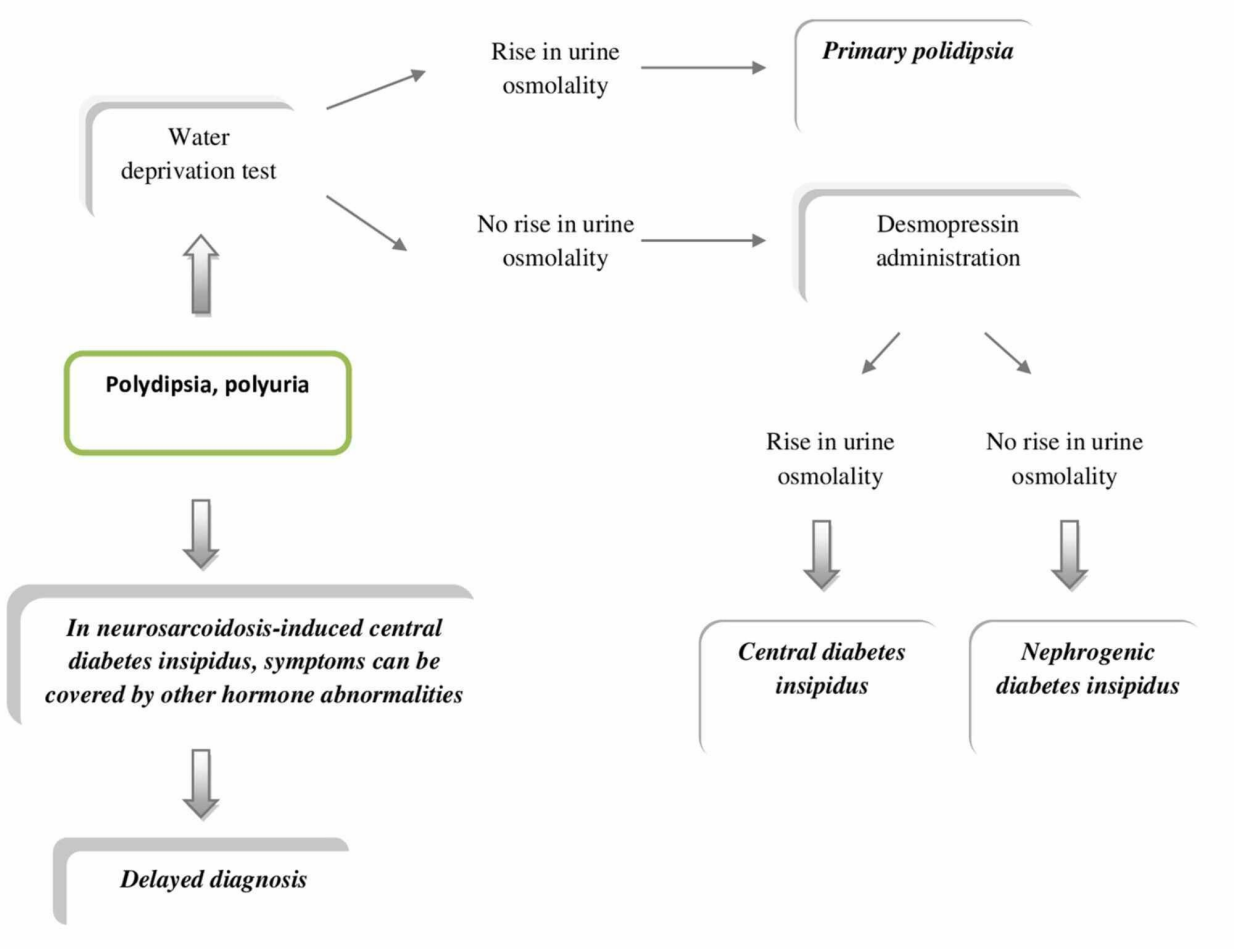
Diagnosis of central diabetes insipidus

The DI diagnostic approach includes history and clinical examination, water deprivation test, and/or desmopressin administration. Water deprivation test can differentiate between primary polydipsia and DI. If water deprivation in a period of eight hours leads to a rise in urine osmolality, a fall in urine volumes diagnosis of primary polydipsia is clear. However, in the case of DI, these findings will not be observed and desmopressin administration is indicated to differentiate between central and nephrogenic DI. If a rise in urine osmolality after desmopressin (ADH analog) administration is observed, central DI is diagnosed since nephrogenic DI is resistant to the effects of desmopressin (Figure 4) [25].

However, assessing the DI in the context of neurosarcoidosis can be difficult which was demonstrated in three interesting cases. A study from 2015 reported the case where symptoms of central DI were covered by adrenal insufficiency which impaired renal free-water clearance. Symptoms of polyuria and polydipsia appeared after steroid replacement therapy [19]. Furthermore, the study from 2016 described a patient who presented with hypernatremia resistant to dextrose 5% water (D5W) therapy with low urine output. After complex diagnostic procedures, subclinical hypothyroidism was revealed which similarly masked the presentation of polyuria in DI. Treatment for this patient included thyroxine along with treatment for hypernatremia which led to the resolution of symptoms. This case showed that thyroid hormone replacement should be considered while evaluating patients with central DI with low urine volumes and persistent hypernatremia despite the administration of desmopressin [18]. In 2020, Solis et al. described a patient who presented with neuropsychiatric symptoms, severe hypernatremia (Na 180 mEq/L), and hypovolemia. Neuropsychiatric symptoms in addition to dysfunction in thirst regulation and significant water deficit masked the typical presentation of polyuria expected in DI [17]. The essential learning point from these studies is that typical symptoms of polyuria and polydipsia expected in DI can be masked by other hormone abnormalities in the case of HP sarcoidosis and detailed evaluation is warranted.

MRI is crucial in diagnosis even though different inflammatory, infectious, and malignant conditions may have similar MRI findings as neurosarcoidosis [35]. The most frequent MRI alterations in central DI due to neurosarcoidosis include pituitary stalk and hypothalamic lesions such as enlarged pituitary with infundibulum thickening, leptomeningeal enhancement in the supra- and infratentorial parts, while some cases reported no MRI abnormalities [18,27,28,31]. An earlier review article that included 20 patients with neurosarcoidosis-induced central DI indicated that symptoms usually fail to improve despite improvement in MRI findings after treatment. Out of 20 patients, 17 showed MRI abnormalities of the hypothalamus and pituitary, and those patients were treated with immunosuppressants. For 14 patients in therapy, MRI findings improved while symptoms of DI persisted [4]. The mean duration from symptoms onset to diagnosis was 3.4 months and remains unclear if a lack of prompt diagnosis and treatment contributed to these findings. One of the possible explanations is that granuloma formation can lead to irreversible damage, particularly when affecting the posterior pituitary gland. According to one hypothesis, it is highly unlikely that sarcoidosis granuloma can completely invade the anterior pituitary because of the tendency of granuloma formation at the base of the brain. Therefore, since the hypothalamus and pituitary stalk are much closer to the base compared to the anterior pituitary, the posterior pituitary can be harder to rescue, and endocrine dysfunction may stay permanent [32].

Moreover, a clinical study that retrospectively reviewed five patients showed that pituitary dysfunction and ADH deficiency persisted in all patients despite therapy which further supports the fact that changes in posterior pituitary due to granuloma formation may be irreversible [29]. Similar findings were observed by Tabuena et al. while following four patients with hypothalamic sarcoidosis for eight years. All the patients responded to corticosteroid treatment with the improvement of symptoms of sarcoidosis, whereas symptoms of central DI failed to correct and long-term treatment with desmopressin was required [30]. A study from 2015 suggested that monoclonal antibody infliximab may be a helpful therapeutic option in HP neurosarcoidosis and can potentially lead to disease remission [37]. Also, spontaneous remission of central DI due to neurosarcoidosis is documented in one case [31].

We can deduce that neurosarcoidosis, although rare, should be included in the differential diagnosis while assessing the cause of central DI. Up to date, central DI caused by neurosarcoidosis is described mostly in case reports and in a few review articles that were all limited by a small number of cases [4,29,30]. In most of these studies, ADH deficiency was permanent. More studies investigating HP changes caused by neurosarcoidosis are essential since there are still no conclusive data if prompt diagnosis and treatment can reverse changes that lead to central DI.

Syndrome of inappropriate antidiuretic hormone secretion in neurosarcoidosis

Pathophysiology

SIADH presents an excess of secretion or action of ADH despite normal serum osmolality and volume. This results in a disorder of water homeostasis and water retention, which leads to hyponatremia with low serum osmolality and high urine osmolality. Regardless of increased total body water, urine volumes are generally unchanged and an acid-base balance is maintained. Edema is typically not seen since the renin-aldosterone system is not activated. Hyponatremia-induced neurological symptoms may vary dependent on sodium levels [39]. The etiology of SIADH can include the ectopic release of ADH, adverse effects of different drugs, or various central nervous system disorders like infections, trauma, and neoplasia [39,40]. Interestingly, neurosarcoidosis, despite more frequently inducing deficiency of ADH, can also be a reason behind the excess production of ADH [15,41-43]. Up to date, we were able to find only four studies in the literature describing sarcoidosis as a definitive cause of SIADH (Table 2).

**Table 2:** Previously published data on SIADH caused by sarcoidosis SIADH: syndrome of inappropriate antidiuretic hormone, ADH: antidiuretic hormone.

Study	Year of publication	Previously diagnosed sarcoidosis
Inappropriate ADH secretion in a patient with systemic sarcoidosis [41]	1992	No
SIADH as presenting feature in a male with coexisting sarcoidosis and systemic lupus [42]	2013	No
A rare presentation of sarcoidosis with SIADH: a case report [43]	2016	No
A case of neurosarcoidosis-induced syndrome of inappropriate secretion of ADH diagnosed with neuroendoscopy [15]	2018	No

Clinical Manifestations and Diagnosis

Assessing the severity of neurological symptoms is crucial when diagnosing a patient with hyponatremia with a sequential decision if emergency treatment with hypertonic saline is recommended [44]. The diagnostic criteria for SIADH include hyponatremia with low serum osmolality, low urine volumes with high urine osmolality, and high urinary sodium excretion without signs of dehydration [40]. For a definitive diagnosis of SIADH, renal failure, adrenal insufficiency, severe hypothyroidism, and nonosmotic stimuli of ADH secretion like pain, nausea, and stress must be excluded [45]. In all previously reported studies, a history of additional symptoms like erythema nodosum, uveitis, cough, and dyspnea, led to a more detailed investigation to determine the primary cause of SIADH [42,43]. MRI findings of the central nervous system included thickening of the floor of the third ventricle and meningeal enhancement [15,42]. Nevertheless, in one reported case, a patient diagnosed with SIADH and subsequently with hydrocephalus had no other symptoms associated with sarcoidosis. Following nodular lesions discovered on MRI of the brain, neuroendoscopic evaluation and biopsy were ordered. The finding of non-caseating granulomas led to the final diagnosis of isolated neurosarcoidosis [15]. This study demonstrates how helpful neuroendoscopy can be in diagnosing and treating neurosarcoidosis-induced SIADH with concomitant hydrocephalus.

The exact mechanism and changes that promote an excess of ADH in neurosarcoidosis are still unclear. Earlier reports suggested that systemic vasculitis can stimulate the HP system and lead to the unregulated secretion of ADH [46]. Furthermore, it is observed that hydrocephalus can impair osmotic regulation and cause SIADH [47]. There is an assumption that similar mechanisms can induce SIADH development in the case of neurosarcoidosis as well, but there is a lack of studies that can confirm this theory [15]. More studies should be performed to justify these implications.

A case report from 2018 demonstrated a different diagnostic challenge in a patient with a history of pulmonary sarcoidosis and severe hyponatremia without a common cause at initial assessment. Comprehensive evaluation excluded SIADH as the cause of hyponatremia, and endocrinologic laboratory tests revealed hypopituitarism. Computed tomography (CT) discovered extensive sellar and suprasellar lesions, which, in light of the history of untreated sarcoidosis, led to the diagnosis of neurosarcoidosis as an underlying cause of hyponatremia [48]. This observation is in line with two additional studies describing hyponatremia cases as the initial presentation of neurosarcoidosis without ADH abnormalities [49,50]. Therefore, even though SIADH as the cause of hyponatremia in neurosarcoidosis has been reported in a few studies, it is uncertain how neurosarcoidosis can lead to hyponatremia in the absence of SIADH. Additionally, cases of neurosarcoidosis-induced hyponatremia without HP involvement have been observed [49]. We can conclude that neurosarcoidosis may present with hyponatremia symptoms with normal levels of ADH; however, the mechanism of this occurrence remains uncertain.

Treatment

The treatment of SIADH includes fluid restriction; however, it is essential to adjust the degree of restriction based on the patient’s capacity for excretion of electrolyte-free water. Continuous monitoring of serum sodium levels, in addition to the assessment of volume status, is crucial [45]. Hypertonic saline infusion is indicated in severe cases of hyponatremia with neurological impairment [44]. Tolvaptan demonstrated its effectiveness in treating SIADH by preventing the effects of ADH at the level of the collecting ducts in the kidneys [15,45]. A previous study suggested that the administration of tolvaptan and other ADH antagonists should be controlled by endocrinologists, nephrologists, and other experienced physicians since clinical experience is limited with these agents. Randomized clinical trials assessing the benefit of vaptans in treating SIADH-induced hyponatremia in contrast to standard therapy are required to provide more evidence and improve patients’ care [45].

In the case of neurosarcoidosis-induced SIADH, the mainstay of treatment is glucocorticoids. In all reported cases, serum sodium levels normalized, and clinical symptoms improved after corticosteroid therapy [15,41-43]. It is essential to highlight that in one case, hyponatremia failed to improve despite recurrent 3% normal saline infusions, and the correction was confirmed after corticosteroid administration [43]. This particular case addresses the importance of correct and prompt diagnosis of sarcoidosis as an underlying cause of SIADH as early treatment can prevent hyponatremia's potentially severe consequences. Therefore, although neurosarcoidosis-induced SIADH is rarely described in the literature, delayed diagnosis and lack of early corticosteroid treatment may lead to persistent hyponatremia and neurological impairment. Since more studies describing the exact pathophysiology and mechanism of how neurosarcoidosis can induce SIADH are required, it is vital to remember that SIADH presenting with severe hyponatremia can be the first clinical manifestation of neurosarcoidosis [15,43].

Importance of early diagnosis and treatment

As mentioned earlier, most hormonal deficiencies remain permanent in the case of HP sarcoidosis [24]. This is demonstrated in a majority of cases with neurosarcoidosis-induced central DI [4,29,30]. In some of the reported cases, the diagnosis was delayed and it is unclear if prompt diagnosis and treatment would prevent these findings [4]. It is indicated that early recognition and steroid treatment can salvage pituitary function since patients may still have partially preserved hormonal axes [32]. Infliximab may be a potential therapeutic option in HP neurosarcoidosis; however, more evidence is needed to support this theory [37]. Glucocorticoids led to the resolution of clinical symptoms and normalization of sodium levels in all reported cases of neurosarcoidosis-induced SIADH [15,41-43]. However, standard therapy of SIADH failed to improve hyponatremia, and the correction was confirmed once corticosteroids were added [43]. Therefore, the lack of prompt corticosteroid administration can lead to severe neurological impairment precipitated by hyponatremia.

Limitation

Our literature review presents several limitations, as a systemic review was not conducted, our paper is subject to bias. Studies included were mostly case reports, case series, and a few review articles and observational studies. We were not able to find any randomized clinical trials related to the topic discussed. In case reports, a certain number of patients were clinically diagnosed with sarcoidosis without histological confirmation. Furthermore, all the aforementioned review articles were limited by small sample sizes. These limitations may cause a flawed comparison of observed findings.

## Conclusions

The HP axis involvement in sarcoidosis can lead to various hormone deficiencies, which may account for the only presenting symptom of sarcoidosis. ADH aberrations are particularly intriguing since sarcoidosis can induce a deficiency, as well as excessive secretion of ADH. Prompt diagnosis of neurosarcoidosis as the cause of hormone abnormalities is crucial since early treatment may presumably rescue pituitary function. However, a diagnosis mostly relies on clinical symptoms and imaging studies, which are not sufficiently specific. Given the limited existing literature on central DI and SIADH caused by neurosarcoidosis, our review will enhance the knowledge we have on this topic.

Besides, by reviewing potential challenges in diagnosing neurosarcoidosis, we aim to help physicians recognize the signs and symptoms early to provide prompt treatment. Physicians should consider neurosarcoidosis as a differential diagnosis when assessing ADH abnormalities. There is also a necessity for developing useful diagnostic tools for early identification of this condition. Considering the rarity of sarcoidosis-induced central DI and SIADH, systemic reviews are unlikely. Further recommendations include multicenter clinical trials investigating therapeutic response with long-term follow-up since implications that early treatment may reverse antidiuretic hormone abnormalities should be justified.
